# Anticancer Activity of New Na(I) Complex on Retinoblastoma Cells via Inhibiting PI3K/AKT/mTOR Pathway

**DOI:** 10.1155/2021/9403333

**Published:** 2021-11-18

**Authors:** Jun Zhang, Zhi-Nan Liu, Guo-Hua Deng

**Affiliations:** Department of Ophthalmology, The Third People's Hospital of Changzhou, Changzhou, Jiangsu, China

## Abstract

Here, through applying 2,6-bis(4′-carboxyl-phenyl)pyridine (H_2_L), a rigid ligand featuring both carboxylic acid and pyridine groups, a new coordination polymer containing Na(I) has been generated with the reaction between H_2_L ligand and NaNO_3_ in a water and DMF mixed solvent, and its chemical composition is [Na_2_L]_n_. Furthermore, the antiproliferative activity of Na(I) complex against the HXO-Rb44 retinoblastoma cells was detected with CCK-8 assay. Hoechst staining along with Annexin V-FITC/PI revealed that Na(I) complex induces the HXO-Rb44 retinoblastoma cells apoptosis. Flow cytometry analysis of reactive oxygen species (ROS) showed that Na(I) complex significantly increases the level of intracellular ROS. Importantly, western blot analysis revealed that Na(I) complex might induce apoptosis through inactivation of PI3K/AKT/mTOR pathway.

## 1. Introduction

Retinoblastoma is the most prevalent intraocular tumor in young children and infants. The incidence is about 1 in 15,000 to 1 in 28,000, with extremely low incidence [1]. However, the current investigation found that the incidence of retinoblastoma is on the rise. At present, with the improvement of living conditions, the incidence of retinoblastoma is increasing year by year [2]. Actually, the retinoblastoma is initiated through biallelic loss of tumor suppressor gene RB1 in more than 95% of cases and develops after additional genetic/epigenetic changes. Importantly, the retinoblastoma is a curable cancer with ocular survival when treated promptly, but it is universally fatal if left untreated [3–5]. It has been revealed that the PI3K/AKT/mTOR pathway plays an important role in the development of the retinoblastoma. Thus, it has been a treatment target of the current research [6].

For the supramolecular structures including the metal in view of crystal engineering, their design and the architecture have caused widespread concern in the coordination and supramolecular chemistry areas. The reason why people pay increasing attention to these areas is on account of their significant unit architecture, at the same time owing to their extensive application prospects in the luminescence, biochemistry along with the catalysis field, especially in the areas of modern pharmaceutical chemistry [7–10]. In the various compounds we have created, the functional complexes have gained extensive interest because of their great pharmaceutical value. As a result, in design of architecture, drug therapy along with the clinical employment, the key factor is to choose an efficient, safe, as well as biocompatible ligand [11–16]. Compared with the complexes on the basis of d^10^ metal ions (known as transition metals), the study of alkaline metal organic complexes is less common, although they process some outstanding advantages such as the following: (a) low toxicity (especially lithium), (b) low cost, (c) high abundance in the earth's crust, (d) stability in the air, (e) low density, providing a weight advantage for the object application, and (f) ionicity, resulting in the strong interactions with the negative functional groups, for example, carboxylates [17, 18]. On the other hand, the nitrogen-heterocyclic carboxylic acid ligands are extensively concerned by the biologists and the chemists on account of their various functional performances and coordination fashions, together with their H-bonding acceptors and donors with the conditions of solution [19–22]. In this experiment, through applying 2,6-bis(4′-carboxyl-phenyl)pyridine (H_2_L), a rigid ligand featuring both carboxylic acid and pyridine groups, a new coordination polymer containing Na(I) has been generated with the reaction between H_2_L ligand and NaNO_3_ in a water and DMF mixed solvent, and its chemical composition is [Na_2_L]_n_ (**1**). Its architecture has been determined via EA, infrared spectroscopy, the diffraction of single-crystal X-ray, TGA, and PXRD. The results of architectural analysis reflected that the Na(I) complex exhibits a three-dimensional skeleton structure on the basis of the one-dimensional rod-like Na-carboxylic acid chains. Besides, the compound **1**'s anticancer activity needs to be explored, and the detail mechanism underlying this effect is still unclear.

Breaking the threshold of ROS balance to induce tumor cell death has been widely used in cancer treatment. It has been reported that many antitumor drugs on the market can induce tumor cell apoptosis by increasing the accumulation of intracellular ROS, such as the doxorubicin and vincristine, which can increase the production of ROS in human promyelocytic leukemia cell lines and bone marrow leukemia cells, respectively, thereby inducing tumor cell apoptosis. The cancer cell has the character of migration and invasion, which was regulated by the signaling pathway of mTOR/AKT/PI3K. Thus, targeting the signaling pathway of mTOR/AKT/PI3K was also a new strategy for the cancer treatment. This research aimed to investigate the anticancer effect and the Na(I) complex's mechanism on HXO-Rb44 retinoblastoma cells. First, CCK-8 assay was employed for the determination of the Na(I) complex's antiproliferation effect against HXO-Rb44 retinoblastoma cells, and then the IC_50_ value of Na(I) complex against HXO-Rb44 retinoblastoma cells was calculated. Cell viability curves suggested that Na(I) complex is an excellent anticancer candidate with low side effects and high activity. Annexin V-fluorescein isothiocyanate (FITC)/propidium iodide (PI) and Hoechst staining assays were then employed to confirm HXO-Rb44 retinoblastoma cell apoptosis. ROS generation assay also verified that the HXO-Rb44 retinoblastoma cell apoptosis was due to the increased intracellular ROS. Whether Na(I) complex could activate the pathway of mTOR/AKT/PI3K, which is significant in cell apoptosis, was also analyzed. The newly synthesized compound has excellent anticancer activity due to its inducing effect on ROS generation and inhibition on PI3K/AKT/mTOR pathway.

## 2. Methods

### 2.1. Chemicals and Measurements

NaNO_3_ (AR) was obtained from Shanghai Guoyao Chemical Group Reagent Company, 2,6-bis(4′-carboxyl-phenyl)pyridine ligand (97%, AR) was supplied by Jinan Henghua Chemical Reagent Company, solvent DMF was accessed from Tianjin Kangkede Chemical Reagent Company, and pure water was produced by Hangzhou Wahaha Group Co., and had the measured resistivity of 18.2 MΩ·cm. PerkinElmer 240C analyzer was exploited for the analysis of hydrogen, nitrogen, and carbon elements. The FT-IR spectra (in the form of KBr pellets) could be harvested through applying the FT-IR spectrophotometer of Bruker Vector 22 with the infrared spectra region from 400 cm^−1^ to 4000 cm^−1^. The diffractometer of Shimadzu XRD-6000 was applied for the patterns of PXRD using the Cu/K*α*radiation (with *λ* of 1.5418 Å) under ambient temperature. The TGA were finished through employing the Netzsch STA449 F1 under flow of air at 10 °C·min^−1^ heating rate in the temperature range of 25–800°C.

### 2.2. Preparation and Characterization for [Na_2_L]_n_

The mixture synthesized from 0.017 g to 0.2 mmol of NaNO_3_, 16.0 mg, and 0.05 mmol H_2_L was dissolved thoroughly into H_2_O and DMF mixed solution (5 mL). The obtaining product was sealed into a 20 mL of reactor lining with Teflon and then it was heated for three days at 140°C. Finally, the product was cooled to environmental temperature; Na(I) complex's colorless crystals were acquired. The yield is 39.2% based on the H2L ligand used. Elemental analysis results (%) for [Na2L]n (formula: C_19_H_11_NNa_2_O_4_): N, 3.86; C, 62.82; and H, 3.05%. Found: N 3.69%, C 62.43%, and H 3.31%. IR (KBr pellet, cm^−1^): 725 (vs), 843 (s), 965 (m), 1239 (m), 1364 (s), 1518 (m), 1619 (s), 1653 (m), 3085 (w), and 3495 (w).

Oxford Xcalibur *E* diffractometer was exploited to acquire X-ray data. CrysAlisPro was utilized for the analysis of strength data, which was then converted to HKL files. The original structural patterns were established and modified through applying direct method-based SHELXS and least-squares strategy-based SHELXL-2014, respectively. The mixture of entire non-H atoms was accomplished by anisotropic parameters. The overall hydrogen atoms subsequently were anchored by AFIX commands geometrically on their adjacent C atoms.  Table 1 reflects the Na(I) complex's data of optimization together with the parameters in crystallography.

### 2.3. Cell Culture

The HXO-Rb44 retinoblastoma cells, ARPE-19, and HepG2 and HeLa cells were all purchased from the ATCC. All the cells were cultured with the DMEM culture medium in the standard condition of 37°C, 5% CO_2_.

### 2.4. Cell Proliferation Assay

The changes of HXO-Rb44 retinoblastoma cells proliferation ability after the treatment of Na(I) complex were measured with CCK-8 Assay Kit (Sigma-Aldrich) adhering to the protocol of manufacturer [23]. HXO-Rb44 retinoblastoma cells were harvested, seeded in six-well plate (Thermo Fisher Scientific) with 1 × 10^6^ cells per well density, and then cultivated in humidified 5% CO_2_ at 37°C temperature. The samples were prepared in triplicate. Then, adding the Na(I) complex with 1, 2, 4, 8, 10, 20, 40, 80, and 100 *μ*M concentrations (100 *μ*L per well), Na(I) complex was firstly solved in the DMSO solution and then diluted into the indicated concentration with PBS solution. The identical volume of oxaliplatin and solvent respectively were employed as the positive and negative control. After incubation for two days, each well of plate was added with 10% solution of CCK-8 and then incubated in darkness for sixty minutes. The microplate reader (Nikon, Japan) was applied to determine the absorbance of each well. All of the investigations were implemented for 5 times. The results of absorbance were calculated and then they were analyzed, and the values of IC_50_ were subsequently calculated. The researches were conducted at least 3 times, and the outcomes were presented as mean ± SD.

### 2.5. Flow Cytometry for Cell Apoptosis

FITC Annexin V Apoptosis Detection Kit I (BD Biosciences, New Jersey, USA) was exploited under the protocol of manufacturer to detect the HXO-Rb44 retinoblastoma cells apoptosis level after incubation with Na(I) complex [24]. The retinoblastoma cells of HXO-Rb44 were harvested and then inoculated with 1 × 10^6^ cells per well density in 6-well plates at 37°C with 5 % CO_2_ in the humidified air. After twelve hours, the cells grew into a logarithmic phase with about 70% density. The wells were added with 2 *μ*M of Na(I) complex and the identical volume of the solvent (negative control) to implement the treatment. After incubation for one day, the cells could be collected and then cleaned for 3 times by utilizing PBS. The HXO-Rb44 retinoblastoma cells were labeled through PI dyes (5 *μ*L) and fluorochrome-binding Annexin V (5 *μ*L). In the end, the flow cytometry (BD Via, New Jersey, USA) was employed for the analysis of all the samples at 625 and 525 nm emission wavelengths and 488 nm excitation wavelength. Each study was carried out for 3 times.

### 2.6. Hoechst Staining Assay

The HXO-Rb44 retinoblastoma cells apoptosis induced by Na(I) complex was further investigated with Hoechst staining assay according to the protocol of manufacturer [25]. The retinoblastoma cells of HXO-Rb44 were collected, inoculated at 5 × 10^5^ cells/well concentration into 6-well plates at 37°C temperature with 5% CO_2_ in the humidified air, and then cultivated by 2 *μ*M of Na(I) complex for one day. Subsequently, discarding the culture medium and cleaning the cells twice through utilizing PBS, they were stained with 0.5 mL of Hoechst solution and then cleaned twice through applying the PBS again. Finally, the cells with blue nucleus were photographed by a fluorescence microscope at 550 nm emission wavelength and 460 nm excitation wavelength to measure the morphological changes.

### 2.7. Measurement of ROS Production by Flow Cytometry

Reactive Oxygen Species Detection Kit was exploited to measure the ROS formation in the retinoblastoma cells of HXO-Rb44 incubated with the Na(I) complex on the basis of protocol [26]. The retinoblastoma cells of HXO-Rb44 were harvested and planted in 6-well plates (2 × 10^5^ cells/well) and then seeded at 5% CO_2_ and 37°C. The wells were added with Na(I) complex (1×IC_50_) and then the wells were incubated for one day. ROSup and PBS were respectively employed as positive and negative control. After that, discarding the medium and cleaning the cells 3 times via the PBS, then the cells were incubated by utilizing H2DCF-DA dye (20 *μ*M) for twenty minutes in darkness. The flow cytometry (BD Via, New Jersey, USA) and FlowJo7.6 were exploited to measure and analyze the absorbance of samples at 488 and 530 nm excitation wavelength. Each research was implemented in triplicate.

### 2.8. Si-RNA Transfection

The synthesized si-RNAs were transfected into cells as previously reported. Briefly, the exponentially growing cells were seeded into 6-well plates and cultured at 37°C in a humidified atmosphere of 95% air and 5% CO_2_. When the cells reach the confluency of 40–50%, the si-RNA transfection was performed with LipofectamineTM3000 Transfection Reagent (Invitrogen, Carlsbad, CA, USA) according to the manufacturer's protocol. The negative group was set transfected with mistake oligonucleotide sequence. Efficiency of gene silencing was detected with western blotting assay.

### 2.9. Western Blot Assay

The mTOR/AKT/PI3K pathway activation in the retinoblastoma cells of HXO-Rb44 was also measured with western blot [27]. After Na(I) complex treatment for twenty-four hours, the lysis buffer containing the PMSF was added into wells. PER™ Mammalian Protein Extraction Reagent (Thermo Fisher Scientific) was exploited to separate their overall proteins, and BCA Protein Assay Kit (Beyotime Biotechnology) was utilized for the calculation of concentration. Protein samples were electrophoresed in the gels of polyacrylamide and then the samples were transferred to the nitrocellulose (NC; Millipore, Burlington, MA, USA) and they were then incubated with corresponding antibodies. 10% bovine serum albumin (BSA; Beyotime Biotechnology) was used to dilute all the primary antibodies, and the dilution ratio is 1 : 1000. The protein expression was determined with p-mTOR, p-AKT, and p-PI3K antibodies (Santa Cruz Biotechnology, Inc.), and ACTIN was exploited as a control.

### 2.10. Statistical Analysis

All the studies were finished at least 3 times, and the obtained data were expressed as mean ± SD. The statistical analysis was accomplished by GraphPad 6.0 (GraphPad, San Diego, CA, USA). The statistical comparisons (*P* values) between more than 3 groups and 2 groups were calculated with one-way ANOVA and student's *t*-test, respectively.

## 3. Results and Discussion

### 3.1. Structural Characterization

**Figure 1:**
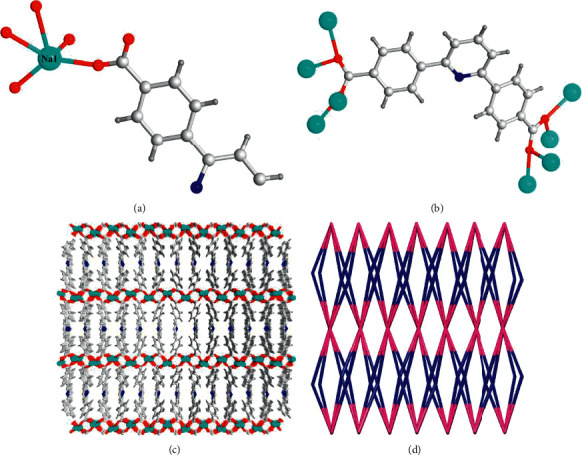
(a) The asymmetrical unit of Na(I) complex. (b) The coordination manner for the ligands of L^2−^. (c) The Na(I) complex's three-dimensional skeleton. (d) The 4,8-linked net topology of Na(I) complex.

Na(I) complex could be generated with the reaction between H_2_L ligand and NaNO_3_ in water and DMF mixed solvent for 72 days at 140°C temperature, which was provided as the colorless crystals, and its chemical formula is [Na_2_L] in accordance with the outcomes from the curves of TGA, EA, and the diffraction of single-crystal X-ray. The optimization outcomes and architectural solution in accordance with the data of single-crystal acquired around environmental temperature indicate that the Na(I) complex belongs to the Pnma space group of orthorhombic system and reveals a three-dimensional skeleton architecture on the basis of one-dimensional rod-like Na-carboxylate chains. The least building unit of Na(I) complex is shown in Figure 1(a), which demonstrates that one Na ion and a half L^2−^ ligand contribute to its asymmetrical unit. The center Na ion is pentacoordinated via 5 oxygen atoms originated from 4 diverse ligands of L^2−^. Interestingly, the five atoms are almost coplanar and such a coordination geometry is less observed in Na(I)-based coordination polymers. In the Na(I) complex, each L^2−^ carboxylic acid group employs the same coordination manners: µ_4_-*η*^2^:*η*^2^ tetradentate connection with four Na ions, which further generate a 1D rod-like Na-carboxylate chains along the *a* axis with the Na-Na separation of 3.471 Å (Figure 1(b)). The Na-carboxylate chains are collected with the ligands of L^2−^ to provide a three-dimensional skeleton architecture with the pyridine N atoms uncoordinated (Figure 1(c)). According to the conclusion of PLATON, there is no solvent accessible volume in the entire skeleton. Topologically, the Na ion and L^2−^ could be respectively regarded as a four-linked node and an eight-linked node, thus the Na(I) complex's entire skeleton could be considered as a (4,8)-linked flu-type network with {4^12.6^12.8^4}{4^6}2 point symbol (Figure 1(d)).

**Figure 2:**
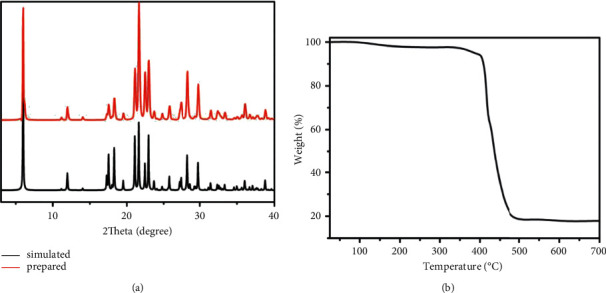
(a) Na(I) complex's PXRD manners. (b) The diagram of TGA curve for the Na(I) complex.

With the aim of testing the products' phase purity, the exploration of PXRD on the complex produced was implemented (Figure 2(a)). For simulated PXRD manners, its peak positions are well consistent with that of experiment results, and this reflects that the crystal architecture is the genuine representative of the whole product of the crystal. For the crystal samples, its selective selection will result in the difference in the strength of product. Simultaneously, the research of thermogravimetric analysis (TGA) was accomplished to explore the Na(I) complex's thermal stability. The Na(I) complex's thermal behavior is detected between 25 and 800°C with 10°C/min heating rate under the flow of N_2_. As shown in Figure 2(b), there is a weightlessness of 2.3% in the range of 170–350°C, which probably was associated with the partly decomposition of the carboxylate groups. When the temperature is higher than 350°C, a second weightlessness could be found, revealing the whole skeleton collapse on account of the organic ligands decomposition.

### 3.2. Antiproliferation Activity of Na(I) Complex on HXO-Rb44 Cells

**Figure 3:**
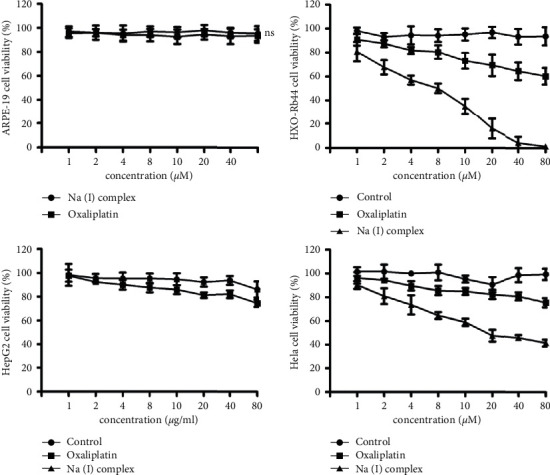
Na(I) complex inhibits the proliferation and growth of HXO-Rb44. HXO-Rb44 retinoblastoma and normal human ARPE-19 cell was treated with serial dilutions of oxaliplatin and Na(I) complex. The Na(I) complex's inhibitory effect on the viability of cell was assessed by CCK-8 assay, and then the curves of cell viability were drawn as control group in contrast to viable cell percentage. The data are described as mean ± SD of 3 separated researches.

The Na(I) complex's antiproliferation activity on the retinoblastoma cells of HXO-Rb44 was measured via CCK-8 assay. The retinoblastoma cells of HXO-Rb44 were incubated with various concentrations of the Na(I) complex for two days, and oxaliplatin was used as the positive control. Cell absorbance, which exhibits the viability of cancer cells after carrying out the treatment, was measured with Epoch microplate spectrophotometer at 570 nm. As exhibited in Figure 3, Na(I) complex reveals remarkable and better antiproliferation effect on HXO-Rb44 retinoblastoma cells compared with the positive control oxaliplatin. The side effect of Na(I) complex was also evaluated on normal human ARPE-19 retinoblastoma cells. Na(I) complex possesses no inhibition on the cell proliferation of ARPE-19. As a result, the results of CCK-8 suggested that the Na(I) complex exhibits potential as a novel anticancer drug having high selectivity and low side effect on the human cancer cells. The inhibitory activity of Na(I) complex against other cancer cells was evaluated as well, and the results showed that the suppression effect of Na(I) complex was much weaker than that against the HXO-Rb44 retinoblastoma cells.

The calculation of Na(I) complex's IC_50_ value was finished in accordance with CCK-8 assay and is presented in Table 2. The IC_50_ of Na(I) complex on HXO-Rb44 retinoblastoma cells is 8.2 ± 0.1 *μ*M and that of positive control oxaliplatin is >80 *μ*M. Thus, Na(I) complex shows high selectivity on human retinoblastoma cancer cells.

### 3.3. Na(I) Complex Induces HXO-Rb44 Retinoblastoma Cell Death through Apoptosis

**Figure 4:**
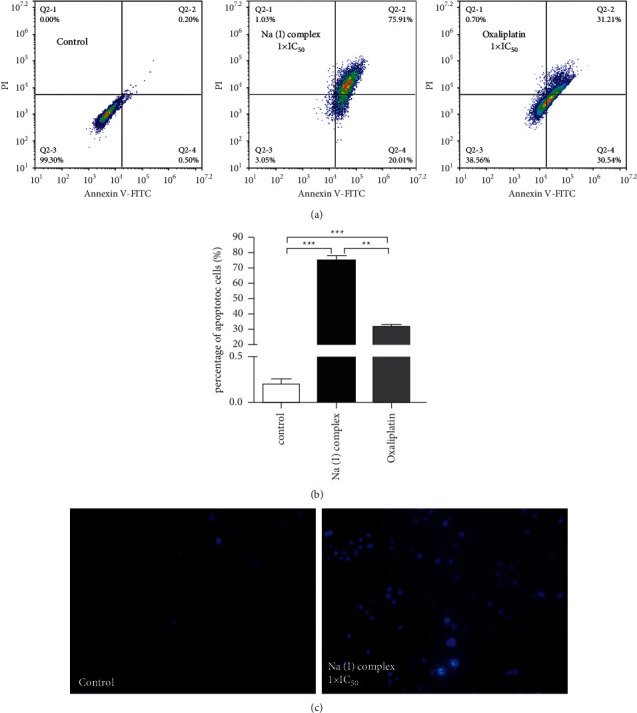
Na(I) complex's effect against the HXO-Rb44 retinoblastoma cells apoptotic induction. The apoptotic cells percentage after the Na(I) complex treatment for one day as determined with a flow cytometer (a). Statistical analysis of (a) (b). Apoptotic cells were stained with Hoechst dye (c). The research was implemented for 3 times, and the outcomes are represented as mean ± SD.

Apoptosis is a type of cell death widely recognized as an important target in cancer treatment. The effect of Na(I) complex on HXO-Rb44 retinoblastoma cellular apoptosis was further studied to investigate the mechanisms responsible for its distinct cytotoxicity. The flow cytometry and Annexin V-FITC/PI double staining were combined to quantitatively analyze Na(I) complex's activity to cause the apoptosis in HXO-Rb44 retinoblastoma cells. As illustrated in Figure 4, Na(I) complex evidently enhanced the early apoptosis of HXO-Rb44 retinoblastoma cells. The apoptosis rate of HXO-Rb44 retinoblastoma cells was 75.91% ± 5.45%, which was obviously greater than the rate in control group (0.2% ± 0.04%). In addition, Hoechst staining was conducted to prove that Na(I) complex could induce HXO-Rb44 retinoblastoma apoptosis.

### 3.4. Na(I) Complex Upregulates Intracellular ROS Generation

**Figure 5:**
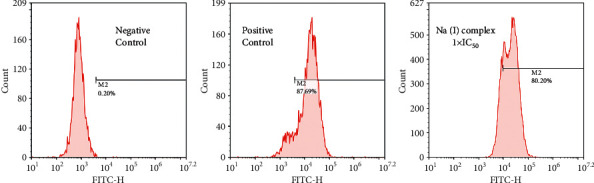
Na(I) complex induces ROS generation in HXO-Rb44 retinoblastoma cells. The retinoblastoma cancer cells of HXO-Rb44 were treated by Na(I) complex and control drug at indicated concentrations, and Reactive Oxygen Species Assay Kit was applied to measure the intracellular ROS in the retinoblastoma cells of HXO-Rb44 by a flow cytometer. The experiment was performed in triplicate.

ROS plays a vital role in HXO-Rb44 retinoblastoma apoptosis and hence was measured through exploiting the flow cytometry. As reflected in Figure 5, the positive control 5-FU could induce the ROS level to 87.69% ± 4.8%, and Na(I) complex shows a similar activity of increasing ROS generation up to 80.20% ± 3.9%. This finding indicates that Na(I) complex induces the cell apoptosis by increasing intracellular ROS.

### 3.5. Na(I) Complex Inactivates PI3K/AKT/mTOR Signaling Pathway in HXO-Rb44 Retinoblastoma Cells

**Figure 6:**
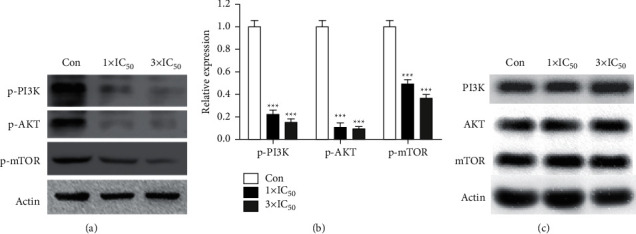
Na(I) complex's effect against the mTOR/AKT/PI3K pathway activation. The retinoblastoma cells of HXO-Rb44 were incubated with Na(I) complex (1×IC_50_) for one day. The cells were collected and then they were lysed, and the p-mTOR, p-AKT, and p-mTOR levels were tested with immunoblotting (a). Densitometry analysis of immunoblotting (b). The mTOR, AKT, and p-mTOR levels were tested with immunoblotting (c). The data are represented as means ± SEM of 3 separated studies; ^*∗*^*p* < 0.05, ^*∗∗*^*p* < 0.01 vs. control.

The canonical signaling pathway of mTOR/AKT/PI3K is a critical regulator of cell proliferation [28]. Given that Na(I) complex could inhibit cell viability and growth, the possible relation of its activity to the signaling pathway of mTOR/AKT/PI3K in HXO-Rb44 retinoblastoma cells was then investigated. Western blot analysis showed that Na(I) complex markedly decreases the phosphorylation levels of PI3K, AKT, and mTOR with the concentration-dependent fashion (Figure 6).

### 3.6. Inactivated PI3K/AKT/mTOR Signaling Pathway Revered the Anticancer Activity of Na(I) Complex in HXO-Rb44 Retinoblastoma Cells

**Figure 7:**
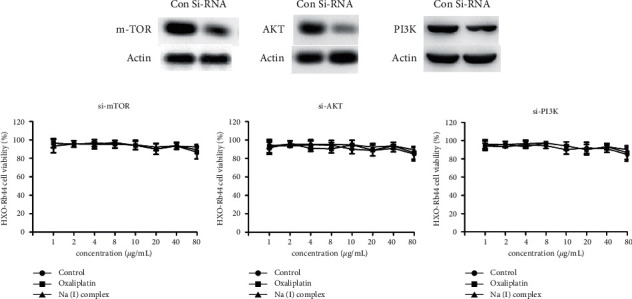
The anticancer activity of Na(I) complex against HXO-Rb44 retinoblastoma cells was abolished by inactivating mTOR/AKT/PI3K pathway. The HXO-Rb44 retinoblastoma cells were transfected with si-RNA, and then Na(I) complex was added for treatment. The viability of the cancer cells was measured with CCK-8 assay.

In Figure 6, we proved that the compound has excellent anticancer activity on the HXO-Rb44 retinoblastoma cells by reducing the activation, which was proved by the reduced levels of p-mTOR, p-AKT, and p-PI3K. To further evaluate the importance of the mTOR/AKT/PI3K pathway, the mTOR/AKT/PI3K pathway was inactivated with si-RNA, and then the inhibitory activity of Na(I) complex on the HXO-Rb44 retinoblastoma cells viability was evaluated with CCK-8 assay. The results in Figure 7 indicated that, after inactivation of the mTOR/AKT/PI3K pathway, the anticancer activity of Na(I) complex against the HXO-Rb44 retinoblastoma cells was also abolished. Through this experiment, the important role of the mTOR/AKT/PI3K pathway in the HXO-Rb44 retinoblastoma cells was further confirmed.

## 4. Conclusion

In this research, we have created a novel coordination polymer containing Na(I) through the reaction between H_2_L ligand and NaNO_3_ in water and DMF mixed solvent. Its architecture has been determined with EA, infrared spectroscopy, the diffraction of single-crystal X-ray, TGA, and PXRD. The results of architectural analysis indicate that the Na(I) complex exhibits a three-dimensional skeleton architecture on the basis of the 1D rod-like Na-carboxylate chains. For the biological evaluation, the anticancer activity and detail mechanism of Na(I) complex were investigated. The **1**'s antiproliferation ability against HXO-Rb44 and the values of IC_50_ were assessed with CCK-8 assay. The curves of cell viability indicate that Na(I) complex has high antiproliferation effect and low cytotoxicity on normal cells, and its anticancer activity is related to cancer cell apoptosis. This compound could induce the percentage of apoptotic cells to 75.91% ± 5.45%, which was remarkably greater than the rate in control group (0.2% ± 0.04%). These data indicated that the Na(I) complex's anticancer effect is mediated through the apoptosis generation. ROS generation and PI3K/AKT/mTOR signaling pathway activation were also detected to explore the mechanism of apoptosis induction. Na(I) complex could induce the ROS generation by inactivating the signaling pathway of mTOR/AKT/PI3K.

In conclusion, Na(I) complex shows potential as an excellent anticancer candidate and has the main effects of antioxidation, anti-inflammatory, anticoagulation, blood lipid lowering, and antiatherosclerosis. Its main anticancer effects have been widely studied mainly from regulating the tumor cell cycle, inducing tumor apoptosis, inhibiting invasion and metastasis, and reversing tumor resistance. Na(I) complex is highly safe with low toxicity and side effects and shows potential as an anticancer drug with therapeutic prospects. The incidence of retinoblastoma in China is still on the rise, so paying attention to the research progress of retinoblastoma treatment drugs should become one of the obligatory tasks of the medical field in our country. Currently, doxorubicin and fluorouracil are the most generally employed drugs for the retinoblastoma treatment. However, the above drugs are too inefficient for the treatment of retinoblastoma, especially for patients with unresectable retinoblastoma, and have no obvious life-prolonging effect. The pretention application values of Na(I) complex provide the clinic a totally new treatment strategy.

## Figures and Tables

**Table 1:** Na(I) complex's data of optimization together with the parameters in crystallography.

Empirical formula	C_19_H_11_NNa_2_O_4_
Formula weight	181.63
Temperature (K)	288.15 (10)
Crystal system	Orthorhombic
Space group	Pnma
A (Å)	6.5632 (2)
B (Å)	29.5529 (10)
C (Å)	8.2174 (3)
Α (°)	90
Β (°)	90
Γ (°)	90
Volume (Å^3^)	1593.86 (9)
Z	4
*ρ*_calc_ (g/cm^3^)	1.514
Μ (mm^−1^)	0.152
Reflections collected	4309
Independent reflections	1854 [*R*_int_ = 0.0171, *R*_sigma_ = 0.0230]
Data/restraints/parameters	1854/0/121
Goodness-of-fit on F^2^	1.056
Final *R* indexes [I ≥ 2*σ* (I)]	*R*_1_ = 0.0420, *ω*R_2_ = 0.0984
Final *R* indexes (all data)	*R*_1_ = 0.0517, *ω*R_2_ = 0.1047
Largest diff. peak/hole/e Å^−3^	0.19/−0.19
CCDC	2087186

**Table 2:** The IC_50_ (*μ*M) values of Na(I) complex for normal and cancer cell lines^*∗*^.

Cell/drug	Na(I) complex	Oxaliplatin
HXO-Rb44	8.2 ± 0.1	>80
HepG2	>80	>80
Hela	20.1 ± 0.23	>80
ARPE-19	>80	>80

^*∗*^Cancer and normal cells were incubated with the sequence of concentrations of Na(I) complex for two days. Oxaliplatin was employed as a positive control. The CCK-8 assay was applied to detect the cell viability, and the value of IC_50_ was calculated. IC_50_ is the concentration of Na(I) complex required to reduce cell viability by 50%. At least three independent experiments were conducted. Each of the value describes as the mean ± SD of 3 separated investigations.

## Data Availability

The data used to support the findings of this study are included within the article.

## References

[1] Lopez A. D., Mathers C. D. (2006). Measuring the global burden of disease and epidemiological transitions: 2002-2030. *Annals of Tropical Medicine and Parasitology*.

[2] Němec L., Fabian P., Tomášek J. (2017). Malignant tumors of the small bowel. *RozhlChir*.

[3] Dimaras H., Corson T. W., Cobrinik D. (2015). Retinoblastoma. *Nature Reviews Disease Primers*.

[4] Dimaras H., Khetan V., Halliday W. (2008). Loss of RB1 induces non-proliferative retinoma: increasing genomic instability correlates with progression to retinoblastoma. *Human Molecular Genetics*.

[5] Zhang J., Benavente C. A., McEvoy J. (2012). A novel retinoblastoma therapy from genomic and epigenetic analyses. *Nature*.

[6] Le Rhun E., Preusser M., Roth P. (2019). Molecular targeted therapy of glioblastoma. *Cancer Treatment Reviews*.

[7] Liao P.-Q., Chen H., Zhou D.-D. (2015). Monodentate hydroxide as a super strong yet reversible active site for CO2capture from high-humidity flue gas. *Energy & Environmental Science*.

[8] Fan L., Zhao D., Li B. (2021). An exceptionally stable luminescent cadmium(ii) metal-organic framework as a dual-functional chemosensor for detecting Cr(vi) anions and nitro-containing antibiotics in aqueous media. *CrystEngComm*.

[9] Wang F., Tian F., Deng Y. (2021). Cluster-based multifunctional copper(II) organic framework as a photocatalyst in the degradation of organic dye and as an electrocatalyst for overall water splitting. *Crystal Growth & Design*.

[10] Fan L., Wang F., Zhao D. (2020). A self-penetrating and chemically stable zinc (ii)‐organic framework as multi‐responsive chemo-sensor to detect pesticide and antibiotics in water. *Applied Organometallic Chemistry*.

[11] Ran H., Du H., Ma C., Zhao Y., Feng D., Xu H. (2021). Effects of A/B-site Co-doping on microstructure and dielectric thermal stability of AgNbO3 ceramics. *Science of Advanced Materials*.

[12] Kang L., Du H., Deng J., Jing X., Zhang S., Znang Y. (2021). Synthesis and catalytic performance of a new V-doped CeO2-supported alkali-activated-steel-slag-based photocatalyst. *Journal of Wuhan University of Technology-Materials Science Edition*.

[13] Zhang J., Zhao J., Sun Y., Xin M., Zhang D., Bian R. (2021). Mechanisms of emerging pollutant Dechlorane Plus on the production of short-chain fatty acids from sludge anaerobic fermentation. *Environmental Science and Pollution Research*.

[14] Xu H., Du H., Kang L., Cheng Q., Feng D., Xia S. (2021). Constructing straight pores and improving mechanical properties of GangueBased porous ceramics. *Journal of Renewable Materials*.

[15] Hu M. L., Abbasi‐Azad M., Habibi B. (2020). Electrochemical applications of ferrocene‐based coordination polymers. *ChemPlusChem*.

[16] Hu M.-L., Razavi S. A. A., Piroozzadeh M., Morsali A. (2020). Sensing organic analytes by Metal-organic frameworks: a new way of considering the topic. *Inorganic Chemistry Frontiers*.

[17] Almáši M., Zeleňák V., Gyepes R. (2020). A series of four novel alkaline earth metal–organic frameworks constructed of Ca(ii), Sr(ii), Ba(ii) ions and tetrahedral MTB linker: structural diversity, stability study and low/high-pressure gas adsorption propert. *RSC Advances*.

[18] Raja D. S., Luo J.-H., Yeh C.-T., Jiang Y.-C., Hsu K.-F., Lin C.-H. (2014). Novel alkali and alkaline earth metal coordination polymers based on 1,4-naphthalenedicarboxylic acid: synthesis, structural characterization and properties. *CrystEngComm*.

[19] Zheng B., Yang Z., Bai J., Li Y., Li S. (2012). High and selective CO2 capture by two mesoporous acylamide-functionalized rht-type metal-organic frameworks. *Chemical Communications*.

[20] Roy P., Schaate A., Behrens P., Godt A. (2012). Post-synthetic modification of Zr-metal-organic frameworks through cycloaddition reactions. *Chemistry - A European Journal*.

[21] Xue D.-X., Cairns A. J., Belmabkhout Y. (2013). Tunable rare-earth fcu-MOFs: a platform for systematic enhancement of CO2 adsorption energetics and uptake. *Journal of the American Chemical Society*.

[22] Song L., Zhang J., Sun L. (2012). Mesoporous metal-organic frameworks: design and applications. *Energy & Environmental Science*.

[23] Tang L. X., Su S. F., Wan Q., He P, Xhang Y, Cheng X. M (2019). Novel long non-coding RNA LBX2-AS1 indicates poor prognosis and promotes cell proliferation and metastasis through Notch signaling in non-small cell lung cancer. *European Review for Medical and Pharmacological Sciences*.

[24] Zhang D., Cao J., Zhong Q. (2017). Long noncoding RNA PCAT-1 promotes invasion and metastasis via the miR-129-5p-HMGB1 signaling pathway in hepatocellular carcinoma. *Biomedicine & Pharmacotherapy*.

[25] Wu J., Gao W., Song Z. (2018). Anticancer activity of polysaccharide from Glehnia littoralis on human lung cancer cell line A549. *International Journal of Biological Macromolecules*.

[26] Song N.-Y., Kim D.-H., Kim E.-H. (2011). Multidrug resistance-associated protein 1 mediates 15-Deoxy-Δ12,14-prostaglandin J2-induced expression of glutamate cysteine ligase expression via Nrf2 signaling in human breast cancer cells. *Chemical Research in Toxicology*.

[27] Gu L.-P., Jin S., Xu R.-C. (2019). Long non-coding RNA PCAT-1 promotes tumor progression by inhibiting miR-129-5p in human ovarian cancer. *Archives of Medical Science*.

[28] Guerrero-Zotano A., Mayer I. A., Arteaga C. L. (2016). PI3K/AKT/mTOR: role in breast cancer progression, drug resistance, and treatment. *Cancer and Metastasis Reviews*.

